# Response of irradiated and bystander cells to charged particles in two-dimensional and three-dimensional colon models

**DOI:** 10.1093/jrr/rrt176

**Published:** 2014-03

**Authors:** Chunjie Li, Hidemasa Kawamura, Saman Khaled, Kathryn D. Held

**Affiliations:** Massachusetts General Hospital/Harvard Medical School, Radiation Oncology, Cox 301, 55 Fruit Street, Boston, MA 02114, USA

**Keywords:** bystander effects, heavy ions, three-dimensional epithelial model, colon cancer

## Abstract

The radiation-induced bystander effect, wherein unirradiated cells near to or sharing medium with irradiated cells express biological responses, most often has been studied in two-dimensional monolayer cultures, although some studies with three-dimensional models and *in vivo* have also shown bystander signaling. We have shown previously that DNA damage, measured as foci of the DNA repair-related protein 53BP1, occurs in unirradiated bystander cells in a three-dimensional skin epithelium model irradiated with protons or iron ions (Lumpkins *et al.*, submitted). In the current work, we extend the studies to a second epithelial model, colon, with studies in both two-dimensional monolayer and a three-dimensional tissue model using Caco2 human colon epithelial cancer cells and AG01522 human fibroblasts.

For the monolayer studies, Caco2 cells in exponential growth were irradiated then co-cultured, sharing medium in an insert system, with unirradiated cells. Cells were irradiated with 250 kVp X-rays at Massachusetts General Hospital or with 1 GeV/amu protons, silicon ions or iron ions at the National Space Radiation Laboratory at Brookhaven National Laboratory. At varying times after irradiation, cell damage was assayed as micronuclei (MN) induction or formation of 53BP1 foci in both irradiated and bystander cells. For the three-dimensional studies, AG01522 fibroblasts were embedded in a collagen gel, then Caco2 cells were grown on the top of the gel. Each three-dimensional construct was cut in half prior to irradiation, with one half irradiated then immediately placed in contact with the other, bystander, half for co-culture. At selected times after irradiation, irradiated and bystander construct halves were fixed and sectioned, and 53BP1 foci were counted.

In monolayer culture, irradiated Caco2 cells showed a dose-dependent increased fraction of cells with MN after exposure to X-rays, protons, iron ions or silicon ions. Bystander Caco2 cells sharing medium with the irradiated cells also showed an increased fraction of cells with MN, reaching similar levels of ∼16% cells with MN, about a threefold increase over controls, after 1 Gy of all types of radiation. The fraction of cells with 53BP1 foci depended on radiation type and time of assay after irradiation, with the induction of foci generally greatest 5 h after irradiation and increasing with radiation dose. In bystander Caco2 cells, the appearance of foci generally was delayed, with the maximal fraction of cells showing foci at 12 h. In three-dimensional culture, after X-ray or proton exposure, cells showed similar trends to those seen in two-dimensional growth, i.e. with both the Caco2 and the AG01522 cells, the fraction of irradiated cells having 53BP1 foci reached a maximum at 5 h, but with bystander cells, the maximum occurred at 12 h after irradiation. This delay in the appearance of foci in bystander cells compared with irradiated cells is similar to our findings in the three-dimensional skin model composed of keratinocytes and fibroblasts.

In summary, our data now show in two different epithelial tissue models in both two-dimensional and three-dimensional models, radiation-stimulated intercellular signaling results in substantial levels of DNA damage in unirradiated cells. Because Caco2 cells are a carcinoma cell line, the studies are now being extended to a three-dimensional colon model using normal human colonic epithelial cells.

